# Data Reliability in a Citizen Science Protocol for Monitoring Stingless Bees Flight Activity

**DOI:** 10.3390/insects12090766

**Published:** 2021-08-27

**Authors:** Jailson N. Leocadio, Natalia P. Ghilardi-Lopes, Sheina Koffler, Celso Barbiéri, Tiago M. Francoy, Bruno Albertini, Antonio M. Saraiva

**Affiliations:** 1 Escola Politécnica, University of São Paulo, Av. Prof. Luciano Gualberto 158, Tv. 3, São Paulo 05508-010, SP, Brazil; balbertini@usp.br (B.A.); saraiva@usp.br (A.M.S.); 2 Centro de Ciências Naturais e Humanas, Federal University of ABC, R. Arcturus 3, São Bernardo do Campo 09606-070, SP, Brazil; natalia.lopes@ufabc.edu.br; 3 Instituto de Estudos Avançados, University of São Paulo, R. Praça do Relógio 109, São Paulo 05508-970, SP, Brazil; sheina.koffler@usp.br; 4 Escola de Artes, Ciências e Humanidades, University of São Paulo, R. Arlindo Bettio 1000, São Paulo 03828-000, SP, Brazil; celso.barbieri@usp.br (C.B.); tfrancoy@usp.br (T.M.F.)

**Keywords:** biodiversity monitoring, data quality, meliponini, protocol validation, volunteer participation

## Abstract

**Simple Summary:**

This work aims to validate a citizen science protocol for monitoring the flight activity of stingless bees. The count of flight activity (entrance, exit, and entrance carrying pollen) filmed in 30 s videos was compared among three different groups: “original” citizen scientists (group that filmed and performed the count in their own videos), “replicator” citizen scientists (group of citizen scientists who performed flight activity counts on videos shot by other citizen scientists), and experts (researchers who work with bees and who performed the counts on videos shot by citizen scientists). The analysis was divided into two levels: perception (detection of activity in videos) and counting. The results of this analysis revealed that citizen scientists and experts have similar perception and count of bee entrance and exit activity, as no statistical differences were found in these two items. However, replicator citizen scientists noticed more bees carrying pollen than original citizen scientists and experts. Despite this, considering only the videos in which the groups agreed on the presence of pollen, the count was similar for both. These results enabled the validation of the protocol and indicated high quality of data produced by individuals who participate in scientific practices following a citizen science approach.

**Abstract:**

Although the quality of citizen science (CS) data is often a concern, evidence for high-quality CS data increases in the scientific literature. This study aimed to assess the data reliability of a structured CS protocol for monitoring stingless bees’ flight activity. We tested (1) data accuracy for replication among volunteers and for expert validation and (2) precision, comparing dispersion between citizen scientists and expert data. Two distinct activity dimensions were considered: (a) perception of flight activity and (b) flight activity counts (entrances, exits, and pollen load). No significant differences were found among groups regarding entrances and exits. However, replicator citizen scientists presented a higher chance of perceiving pollen than original data collectors and experts, likely a false positive. For those videos in which there was an agreement about pollen presence, the effective pollen counts were similar (with higher dispersion for citizen scientists), indicating the reliability of CS-collected data. The quality of the videos, a potential source of variance, did not influence the results. Increasing practical training could be an alternative to improve pollen data quality. Our study shows that CS provides reliable data for monitoring bee activity and highlights the relevance of a multi-dimensional approach for assessing CS data quality.

## 1. Introduction

Citizen science (CS) is a scientific approach that allows members of the general public to contribute to the scientific process, usually as data collectors but desirably in other scientific inquiry steps [1,2]. As data contributors, the participants can provide large amounts of data that, otherwise, would require great time availability, as well as substantial financial resources [3,4]. This scattered and diverse information can reduce data scarcity problems, which are common in some study fields (e.g., species populations distribution, water quality monitoring). Albeit still underutilized, CS can be a valuable resource for global change research and UN Sustainable Development Goals achievement [3,5].

Data quality (DQ), which can be considered a multi-dimensional issue [6], is a concern for several researchers involved with CS activities [7,8]). Studies report on different dimensions of data quality, such as standardized sampling (e.g., [9]), spatial and temporal representativeness and bias (e.g., [10]), data accuracy and precision [8,11,12], sample size (e.g., [13,14,15,16]), volunteers’ proper training (e.g., [17,18,19]), and the experience and ability levels of participants [20,21].

In this sense, the work developed by Wiggins and Crowston [2] analyzed the mechanisms for data quality assurance in 128 CS projects and concluded that the topic is a concern in most of them. The most common procedures to ensure data quality observed were: expert review, photo submissions, paper data sheets submitted along with online entry, replication or rating by multiple participants, quality assurance/quality control (QA/QC), training programs, and automatic filtering of unusual reports. Pilot-testing of citizen science protocols is also a strategy commonly used to improve data quality and reliability. The feedback from testing participants is essential to redesign the protocols and build appropriate materials for the project [22].

Strategies for DQ control may be applied during data collection, data classification, or data analyses through statistical and modeling tools [8]. Data quality control during classification, for instance, may include replication among volunteers (distinct volunteers performing the same task and reaching some consensus—cross-comparisons) and expert validation (comparing citizen scientists’ data with professional scientists’ data) [23]. However, few studies have systematically tested these control mechanisms (e.g., [16,24,25,26]. Aceves-Bueno et al. [27], for instance, analyzed 63 citizen science papers that reported 1363 observations of expert validation and found that 73% of the abstracts described the contributions of citizen science as positive (accurate, reliable, comparable, statistically similar, or valuable) and only 13% assessed citizen scientists’ (cs) performance negatively (no significant correlations, overestimated, or contradictions). In addition, validated CS data was reported to be more cost-effective than traditional methods [15].

In a review of citizen science initiatives with bees, Koffler et al. [28] reported the use of various strategies related to data quality assessment and control, mainly digital vouchers (photographs submitted by citizen scientists) (43.2%), expert review of data (40.9%), use of structured protocols (40.9%), and training of participants (29.6%). The same initiative used up to five different strategies, indicating that data reliability was a major concern for the projects’ teams. For instance, data quality was stated as the primary objective by 13.6% of the 88 studies analyzed, with protocols mainly related to sampling effort and species identification. While bumblebees and honey bees were the most investigated groups, only three works studied stingless bees, despite the increasing interest in this group due to their importance as pollinators [29] and the global expansion of meliponiculture activities [30,31]. Stingless bees comprise a diverse group with more than 500 recognized species in tropical and subtropical regions [32] and stingless beekeeping may be an important tool for sustainable rural development and conservation [33]. However, the lack of basic knowledge of stingless bee ecology and management still hampers the practice [31,34]. In this context, beekeepers may act as important partners in CS projects with stingless bees, following successful ongoing initiatives with honey bees [35]. Monitoring stingless bees’ flight activity, for instance, can help us understand several factors that affect colony performance, such as responses of foraging bees to intra-colony stimuli and meteorological conditions. Flight activity data also serve as an economic evaluation of the colony since the number of foraging trips is directly linked to colony production and pollination services [36]. Therefore, good quality data production is essential to subsidize management strategies.

The present study aimed to assess the data reliability of a structured citizen science protocol for monitoring stingless bee flight activity. Our initial hypothesis is that there are no statistical differences between the data produced by citizen scientists in comparison to data produced by experts, although the dispersion of the data produced by citizen scientists may be greater. Participants of an outreach course related to citizen science and meliponiculture produced the data. Original data gathered by citizen scientists was first replicated by a group of citizen scientists, who also participated in the project, and then validated by a group of experts. We tested data accuracy for replication (comparing original and replicated data) and for expert validation (comparing citizen scientists and expert data). Moreover, precision was analyzed for data validation, comparing dispersion between replicator citizen scientists and expert data. Since flight monitoring is a task with considerable difficulties for untrained personnel, two distinct activity levels were considered: (a) perception of flight activity (whether the activity was detected or not) and (b) flight activity (bee counts when activity was detected). Our approach provides a multi-dimensional assessment (accuracy and precision in perception and counts) of reliability in citizen science data for a non-model insect organism.

## 2. Material and Methods

**Figure 1:**
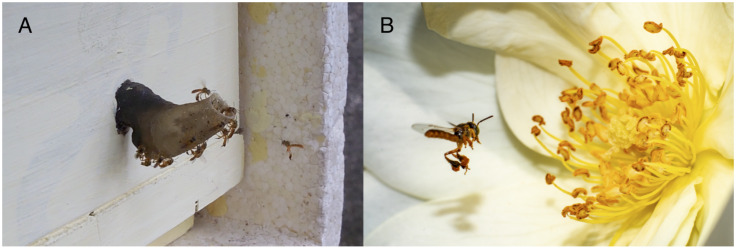
(**A**) A frame of one of the received videos showing a bee approaching the nest entrance tube and some guard bees. (**B**) An image of a bee carrying pollen attached to its hind legs. Photo by André Matos.

During an outreach course on meliponiculture and citizen science held in July 2020, participants were invited and trained to perform and pilot-test a structured protocol aimed at the monitoring of flight activity for *Tetragonisca angustula* (Latreille, 1811), a stingless bee widely distributed in Brazil and commonly reared by beekeepers across the country. The protocol stated that citizen scientists had to film the nest’s entrance for 30 s within different time intervals (between 7:00 a.m. and 9:00 a.m.; 11:00 a.m. and 1:00 p.m.; 3:00 p.m. and 5:00 p.m.). Data collection consisted of watching the videos and counting how many bees entered (entrance), how many left the nest (exit), and how many came in carrying pollen (pollen) in that period (Figure 1A). As stingless bees carry pollen attached to the pollen baskets in the hind legs (Figure 1B), pollen loads were visible and could be identified in video recordings. Data submission was carried on a web system developed exclusively for this purpose (https://beekeep.pcs.usp.br, in Portuguese, University of São Paulo, São Paulo, Brazil—access date: 23 August 2021), also collecting other relevant variables for further studies.

**Figure 2:**
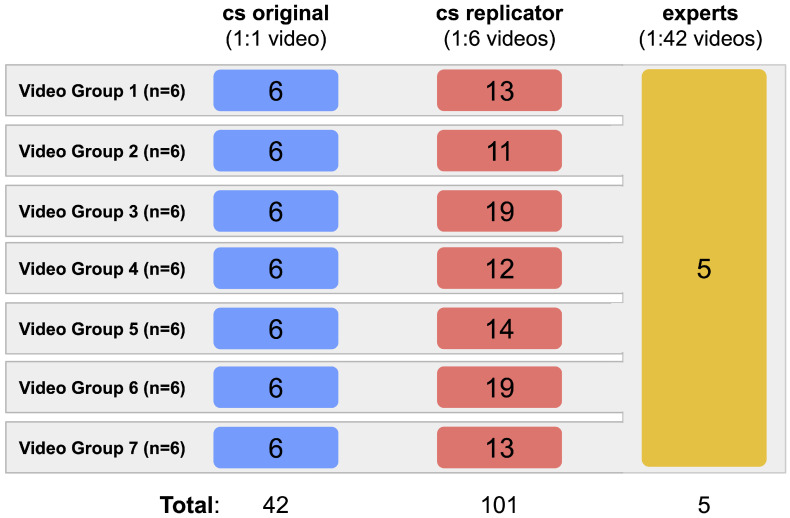
Distribution of the 42 videos for each group: cs original (*n* = 42 individuals—1:1 video), cs replicator (*n* = 101 individuals—1:6 videos) and experts (*n* = 5 individuals—1:42 videos).

Of more than 400 submitted videos, 42 were randomly selected for this study purposes, along with the counts (Supplementary Material 1) provided by citizen scientists at the time of video submission (these participants are hereafter called “cs original”). The videos were divided into seven groups of six videos each. For the replication analyses among citizen scientists, each group of videos was assessed by at least 11 participants from a total set of 101 citizen scientists (from now on called “cs replicators”), none of them included in the cs original group. Group size varied from 11 to 19, none of them analyzed videos of more than one group. For the validation process, a set of five experts in stingless bee behavior analyzed all videos, including three authors of this study. Thus, at the end of the quality control process, there were 2574 countings (considering 858 countings both on entrances, exits, and pollen), 126 of which were from the cs original (42 individuals × 1 video per individual × 3 countings per video), 1818 from the cs replicators (101 individuals × 6 videos per individual × 3 countings per video), and 630 from experts (5 individuals × 42 videos per individual × 3 countings per video) (Figure 2).

### Data Analysis

The groups (cs original, cs replicators, and experts) and video quality were considered potential sources of observed differences. In order to assess video quality, four variables were assembled through a Principal Component Analysis (PCA): Mean Structural SIMilarity (MSSIM), Focus, Contrast, and Frames per Second (FPS). MSSIM was obtained using a custom script that relies on OpenCV 4.5.1 implementation of MSSIM as described by Wang et al. [37]. We also considered other traditional metrics such as MSE (Mean Square Error) and the correlated PSNR (Peak Signal-to-Noise Ratio), but MSSIM is a better metric when considering the human perception of the video quality [38]. Focus and contrast indicate qualitatively if the nest was in the foreground and if it was possible to differentiate the bees from the background, respectively. These two metrics were determined by a designer specialist, who watched the videos and checked both attributes in each. FPS indicated the number of frames per second and was extracted from video metadata or inferred using the file size and the video bitrate (uncompressed) when the metadata was missing or wrong (e.g., recorder used variable bitrate). Before performing the PCA, FPS data were scaled by subtracting each value from the mean and dividing it by the standard deviation. PCA Axis 1 explained 68% of the data variability and was used as a proxy for video quality in our analyses.

Generalized Linear Mixed-Effects Models (GLMER) were adjusted to analyze accuracy for replication and validation processes. A Boolean variable was created to indicate the presence (when greater than zero) or absence of activity and to assess the probability of flight activity perception. The perception of flight activity was modeled as a binary response (presence—when the participant responded with a value greater than zero for the specific activity—or absence of activity), following a Bernoulli distribution. At the same time, the effective counts of activity were modeled using a Poisson distribution (using only data for which counts were greater than zero). The group was included in the model as a fixed effect (cs original compared to cs replicators for replication analyses; cs replicators compared to experts for validation analyses). The video quality was included in the models as a covariate. A random effect was set for the videos to account for dependencies in the data, with participant identity nested within each video. Initially, complete models with response variables for each bee activity were set (entrances, exits, and pollen). Then, reduced models were adjusted by removing the fixed effects, one by one, until the null model. Likelihood-ratio tests were employed to compare models and to select the best model in each analysis. Overdispersion was assessed by verifying the data’s standard deviation (sd) against the sd of simulated data. The estimated coefficients were back-log-transformed using the exponential function to obtain the values of odds or odds ratio from the models.

The Median Absolute Deviation (MAD) was used as an indicator of counting dispersion for both the group of cs replicators and the group of experts. All the counts were considered in this analysis, even when no activity was found (zero values). Data from cs original could not be included in this analysis, as no replicates were performed, and hence no variation could be measured. Like other studies that analyzed count-based protocols, we chose the median-based metric because data were non-parametric, and the median minimizes the influence of some extreme countings [16,39]. The mean of the MAD of each video was calculated for each group and compared through a paired Wilcoxon signed-rank test to verify possible statistically significant differences between the groups. This non-parametric test was used because the paired differences in MAD values between groups were not approximately normally distributed. We also analyzed the correlation between the mean of MAD and the median of the activities counts to assess whether there was a relationship between bee activity rates and variation in counts in our protocol, using the Pearson correlation coefficient.

All analyses were performed in R (version 4.0.4) [40], employing the functions and respective packages: scale (base), prcomp (stats), GLMER (lme4), anova (stats), mad (stats), wilcox.test (stats), cor.test (stats) and testDispersion (DHARMa).

## 3. Results

### 3.1. Perception and Effective Countings

**Figure 3:**
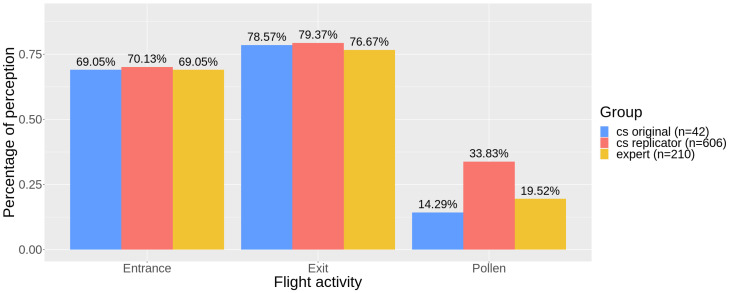
Absolute frequency of perception of activity (entrance, exit, pollen) by the groups (cs original—in blue —, cs replicator—in red—, expert—in yellow) (see also Figure 2).

**Figure 4:**
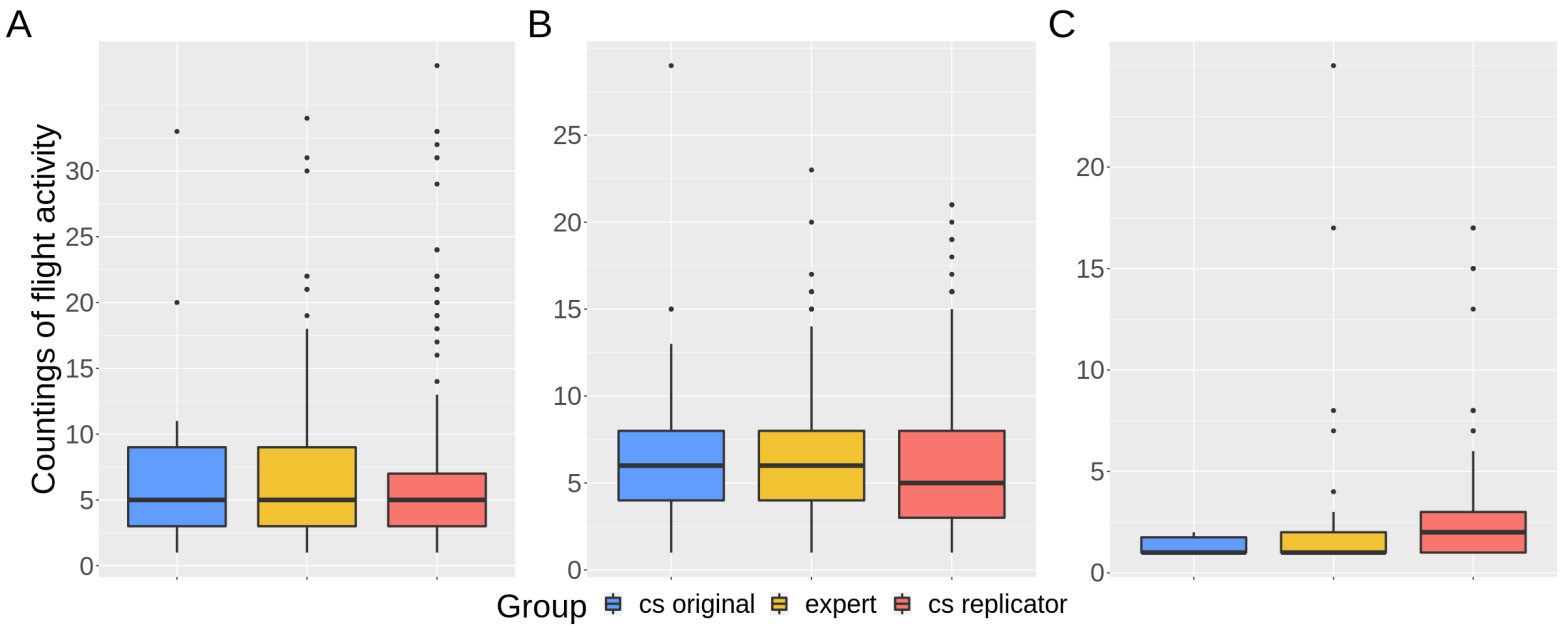
Medians and dispersion of effective countings for: (**A**) entrances; (**B**) exits; (**C**) pollen, performed by cs original (in blue), cs replicators (in red), and experts (in yellow). The dots are outliers, countings over or under 1.5 times the interquartile range.

The perception of entrance activity from the videos ranged from 69% (cs original and experts) to 70% (cs replicators), while the perception of exit activity ranged from 76% (experts) to 79% (cs replicators). Perception of pollen was much lower than the previous activities and varied from 14% (cs original) to 34% (cs replicators) (Figure 3). For those videos with perceived activity, the median for entrance counts was 5 for all the groups; medians for exit counts were 6, 5, and 6, while medians for pollen counts were 1, 2, and 1 (for cs original, cs replicators, and experts, respectively) (Figure 4).

### 3.2. Accuracy

Regarding replication analyses (comparison between cs original and cs replicator), the likelihood-ratio tests indicated the null models as the best ones for the perception of bees entering and leaving the nest (Table 1). However, for pollen perception, group effect was found. In this case, cs replicators presented odds increased by a factor of 4.63 compared to cs original (Table 2). The null models were the best also for the counts of entrance, exit, and pollen (Table 1). These results indicate that both groups performed countings quite similarly. Video quality did not present any effect in these analyses.

In the validation analyses (comparison between cs replicators and experts), null models were the best ones for the perception of entrance and exit and for the counts of entrance, exit, and pollen (Table 1). However, for pollen perception, the best model included the group variable, where the cs replicators had odds of perceiving pollen increased by a factor of 2.87 when compared to experts (Table 2). Here, the video quality index also did not add relevant information to explain the participants’ data. Even though the model for pollen counts presented significant overdispersion despite adjustments (Supplementary Material Table S1), i.e., the residual variance was larger than expected under the fitted model, no effect of the tested variables was found.

### 3.3. Precision

**Figure 5:**
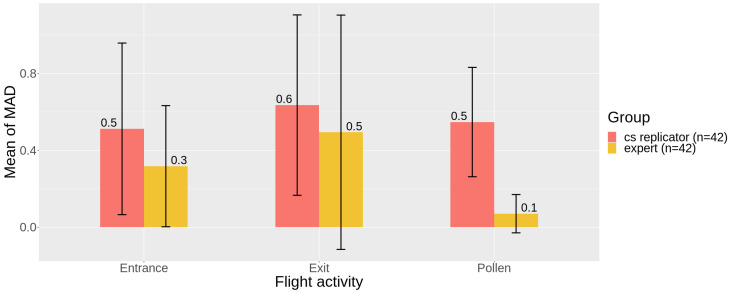
Mean of Median Absolute Deviation (MAD) of all videos for entrance, exit, and pollen counts, for cs replicators (in red) and experts (in yellow). Vertical bars indicate the Confidence Intervals (95%).

The MAD means were statistically equal between cs replicators and experts for entrance (V = 24.5, *p*-value 0.071) and exit (V = 54, *p*-value 0.052) counts. However, the MAD mean was higher for pollen counts for cs replicators in relation to experts (V = 85, *p*-value 0.005), who exhibited low dispersion for this task (Figure 5 and Supplementary Material Figure S1). These results are in accordance with the accuracy analyses, which presented significant differences for pollen perception between groups.

**Figure 6:**
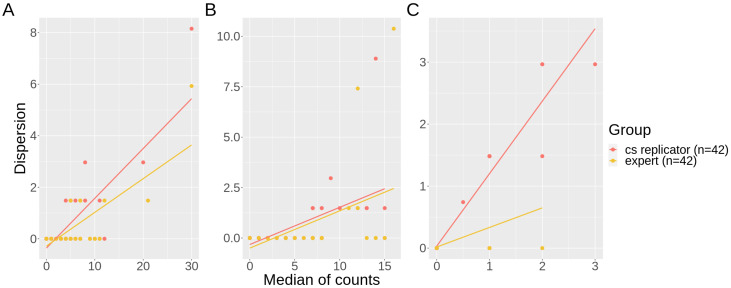
Correlation between the median of counts and dispersion (MAD) values of each video by group (cs replicator in red and experts in yellow), with tendency line (linear model line). (**A**) entrances; (**B**) exits; (**C**) pollen.

Data dispersion for the countings was positively correlated with the amount of bees in activity in the videos: entrance, cs replicators (r = 0.8, *p*-value < 0.001) and experts (r = 0.78, *p*-value < 0.001); exit, cs replicators (r = 0.57, *p*-value < 0.001) and experts (r = 0.46, *p*-value 0.002). For entrance and exit, the pattern of increasing dispersion is similar for both groups, however, for pollen, the correlation is stronger for citizen scientists: cs replicators (r = 0.94, *p*-value < 0.001) and experts (r = 0.43, *p*-value 0.004) (Figure 6).

## 4. Discussion

Count-based activities are tasks with low to medium skill or training requirements [8]. In the present study, our results evidenced that, depending on the task, the accuracy of different groups can be affected. This was especially true when the perception of bees carrying pollen was considered. Thus, specific ability levels may be necessary to guarantee reliability in distinct contexts. For instance, perceiving a moving bee is significantly different from perceiving pollen present at a bee corbicula, which is a much smaller target and may require more volunteer training to reach the same level of quality of the experts. In the work of Bieluch et al. [41], CS program coordinators were interviewed about the contribution of volunteers in fish count-based protocols. They highlighted some aspects that can influence the counting accuracy, like the poor weather, high numbers of individuals passing at once, and the physical attributes of the counting site. Here, attributes of the video, which could be a proxy for context variation, did not affect countings. Target size may also influence perception, and large individuals and distinctive shapes can help to produce more accurate data [39]. It could pose as a barrier for data quality in our study since the size of the studied bees is small, varying between 4 mm and 5 mm, imposing some difficulty for those who are analyzing the videos [42]. Finally, stingless bees normally speed up when near the nest entrance, which could also hinder the perception of bees carrying pollen by citizen scientists [43].

The observed divergence in pollen perception by cs replicators compared to the other groups cannot be fully explained in this study, where experts presented conservative counts, while cs replicators exhibited a permissive performance. We hypothesized that due to citizen scientists’ eagerness to find some pollen and their lack of experience, they could overestimate the perception of this activity. If this is true, with more practical training effort in this protocol, we expect that cs replicators would present lower and less variation in the perception of pollen loads [44]. Some questionable research practices, such as falsification (wilful or unintended distortion of data or results), can negatively impact research [45], so they must be seriously considered when resulting from citizen science practices.

Generally, false negatives are a concern for researchers that deal with biodiversity occurrence data from citizen science (e.g., [46,47], but false positives are generally considered unimportant, although they can lead to severe biases in conclusions about ecological systems [48]. Overestimation and underestimation of counts in citizen science protocols are reported in the literature. For instance, citizen scientists underestimated experts’ countings of seals and sea lions in static images [39], countings of stomata and epidermal cells in static images [49], and the estimation of caterpillar density [50]. On the other hand, they overestimated fly and beetle density when applying a visual survey protocol in natural habitats [50]. These results varied according to the protocol and factors related to the individuals, such as experience and training [51]. However, empirical evidence suggests that citizen science data quality has often been sufficient for the projects’ aims, and differences between citizen scientists’ and professionals’ data were not significant in most cases, e.g., 61.6% of studies showing no significant differences between citizen scientists and professionals in Aceves-Bueno et al. [27].

Considering the count precision in our study, intra-group dispersion was found in both groups, which is naturally expected in science, regardless of the individuals’ expertise. For instance, in Swanson et al. [16], during an expert verification, precise counts of specimens were unresolvable in many of the images they were analyzing, and the specialists agreed on the number of individuals only 74% of the time. The authors concluded that multiple citizen scientists classifying an image could be more reliable if compared to a single person, even if this person is an expert. In our study, when more bees were in flight activity, more dispersion was found in all groups, which can reveal a greater difficulty for counting many bees at the same time. Other confounding factors could inflate countings, such as the presence of guards at the nest entrance. These guards are larger and heavier than the regular workers, hovering or standing next to the nest entrance tube (Figure 1A) [52], possibly being confused with bees in foraging activity when activity is high. We also found that CS data, specifically for pollen counts, showed higher dispersion than expert data. Likewise, data variability among citizen scientists was tested in Fehri et al. [53], in which the volunteers were engaged and trained on using rain gauge tools. Data dispersion was slightly higher in specific situations (high rainfall events), but in contrast, other events measurements (lower precipitation) showed more consistency for the group composed of citizen scientists. More straightforward tasks performed by volunteers tend to present less data dispersion when compared to more complex ones [53,54]. Additionally, volunteers generally improve their accuracy as they gain experience within a project [8]. The continuous execution of a task can promote personal learning and progress of the required skill [44] and improve data quality [27]. In our citizen science initiative, the participants of the outreach course learned to perform the protocol based on three video lessons and online guidance since any practical activity was not possible due to the restrictions imposed by the COVID-19 pandemic. Therefore, it is possible that an increased effort in the training of volunteers could lead to higher quality data in the present study, as has already been reported for other citizen science studies (e.g., [19,55,56]). Clear and objective protocols can also help in the rigorous collection of data [23,57].

Here, replication was used as a way to test for data quality, which is a practice reported to produce high rates of accuracy [16,39,58], since the combination of different and independent contributions decreases the errors observed individually in each one, according to the “Wisdom of Crowds” principle [59]. This quality assurance procedure can be used by default in the protocol design by proposing that all the collected data should be confirmed by a set of volunteers, or only in cases of unusual records, for example. A challenge with this approach is to define the value of agreement between individuals [39], sometimes with the support of expert checks. The level of accuracy needed will likely depend on the research question and the ability to perform post hoc statistical manipulation on these data [54]. In our case, replication provided reliable CS data for the counts of bees entering or leaving the nest. However, the perception of pollen was consistently different between groups (Figure 3, Table 1 and Table 2) and countings were also highly variable for citizen scientists (Figure 5 and Figure 6). Thus, although effective counts of pollen were reliable, both in replication and validation analyses (Figure 4), the observed variance in the replicators’ data can impose some data quality issues and compromise the application of these specific data.

As a consequence of these results obtained for pollen in the present pilot test of the protocol, a checkbox option labeled “pollen count was performed” was included in the platform, making it possible for citizen scientists to report an actual absence of pollen activity (“real” zeros) differently from the zeros that represent that they were unable to perform the task. Indeed, including an “I don’t know” option was shown to enhance data quality and contribute to the agreement among participants in a citizen science initiative [60]. In addition, we realized the importance of slowing down the video speed to make pollen loads easier to see. Thus we have also included this functionality in our platform as well. Future works may include developing an agreement algorithm to determine the consensual entrance, exit, and pollen activity in each video and the use of artificial intelligence to perform automatic counts.

## 5. Conclusions

Our results indicate that the flight activity protocol for stingless bees provides reliable data for bees entering and leaving the nest since original, replicated, and expert data were similar. These results are in accordance with our initial hypotheses that CS data do not differ statistically from those provided by specialists. However, a significant difference was found for the perception of pollen loads, with the cs replicator diverging from the experts and the cs original. Despite that, for those videos in which there was an agreement about pollen presence, i.e., in which both groups identified bees carrying pollen, the effective counts were very similar and confirmed the reliability of CS-collected data.

Data quality is a common concern in the era of data, and mechanisms to evaluate and improve quality are essential to ensure data applicability in its intended purpose. In CS, the problems are, in fact, comparable to those found in the traditional scientific exercise [3,8] and both models need to implement specific measures to guarantee the data quality and the other obtained products [54,61]. In CS, these quality requirements can be research questions of interest to participants, viable protocols, consistent training, evidence of observations, replication, expert review, among others. Several studies in different fields of knowledge, including the present one, report positive results and highlight characteristics that justify the investment in volunteers to act as scientists [12,27,62,63]. Depending on the particularities of the required task, adequate training and specific skill levels may be necessary, as in any other scientific approach. Our study highlights the importance of a multi-dimensional approach in CS data quality assessments to identify potential pitfalls and adequate protocols to improve data collection and use of CS data in research.

## Figures and Tables

**Table 1:** Likelihood-ratio tests for model selection in the replication (comparison between cs original and cs replicator) and validation (comparison between cs replicators and experts) analyses. $\chi^{2}$ statistic, with the respective degrees of freedom (Df), and *p*-value for each test, are presented. Significant differences between models are followed by an asterisk.

		Response	Starting Model	Fixed Effect Removed	$\chi^{2}$	Df	*p*-Value
Replication	Perception	entrance	group + video quality	group	0.156	1	0.693
			video quality	video quality	0.045	1	0.832
		exit	group + video quality	group	0.356	1	0.551
			video quality	video quality	0.065	1	0.799
		pollen	group + video quality	group	10.852	1	0.001 *
			group + video quality	video quality	0.064	1	0.801
			group	group	10.857	1	0.001 *
	Count	entrance	group + video quality	group	1.674	1	0.196
			video quality	video quality	0.003	1	0.957
		exit	group + video quality	group	0.658	1	0.417
			video quality	video quality	0.001	1	0.981
		pollen	group + video quality	group	1.367	1	0.242
			video quality	video quality	0.063	1	0.802
Validation	Perception	entrance	group + video quality	group	0.516	1	0.472
			video quality	video quality	0.056	1	0.812
		exit	group + video quality	group	0.592	1	0.442
			video quality	video quality	0.003	1	0.958
		pollen	group + video quality	group	22.325	1	0.001 *
			group + video quality	video quality	0.077	1	0.781
			group	group	22.330	1	0.001 *
	Count	entrance	group + video quality	group	0.039	1	0.843
			video quality	video quality	0.038	1	0.845
		exit	group + video quality	group	0.035	1	0.851
			video quality	video quality	0.001	1	0.981
		pollen	group + video quality	group	0.315	1	0.575
			video quality	video quality	0.219	1	0.640

**Table 2:** Parameter estimates of the final models for pollen perception in replication and validation analyses.

Model	Comparison	Predictor	Estimate	SE	Pr(>|z|)	Odds/Odds Ratio
Pollen perception	Replication	cs original (intercept)	−2.7561	0.333	<0.001	0.06
		cs replicators	1.5332	0.508	0.003	4.63
	Validation	experts (intercept)	−2.3004	0.344	<0.001	0.1
		cs replicators	1.0529	0.229	<0.001	2.87

## Data Availability

The raw data used in this study are provided as Supplementary Material.

## Supplementary Materials

The following are available online at https://www.mdpi.com/article/10.3390/insects12090766/s1, Supplementary Material 1: Excel file with complete data-set, containing citizen scientists original (cs-original tab), replicated (cs-rep tab), and expert (exp tab) counts, along with video quality metric; (vq tab); Supplementary Material 2: Table S1: Dispersion of residual variance for each model. Significant dispersion models are highlighted in bold, Figure S1: Median Absolute Deviation (MAD) value of each video for replicator citizen scientists and experts in different bee activities.

## Abbreviations

The following abbreviations are used in this manuscript:

CS	Citizen Science
cs	Citizen Scientist
Df	Degrees of Freedom
DQ	Data Quality
FPS	Frames per Second
GLMER	Generalized Linear Mixed-Effects Model
MAD	Median Absolute Deviation
MSE	Mean Square Error
MSSIM	Mean Structural SIMilarity
PCA	Principal Component Analysis
PSNR	Peak Signal-to-Noise Ratio
QA	Quality Assurance
QC	Quality Control
sd	Standard Deviation
SE	Standard Error
