# Supplementary material for: Data Reliability in a Citizen Science Protocol for Monitoring Stingless Bees Flight Activity
Source: Insects. 2021 Aug 27;12(9):766. doi: 10.3390/insects12090766 (PMC8467663; doi:10.3390/insects12090766)
Supplement: Supplementary file 1 [file insects-12-00766-s001.zip › insects-1302791-supplementary/MDPI__Supplementary_Materials_2.pdf]

Supplementary Materials: Data reliability in a Citizen Science protocol for monitoring stingless bees flight activity

Jailson N. Leocadio [1], Natalia P. Ghilardi-Lopes [2], Sheina Koffler [3], Celso Barbiéri [4], Tiago M. Francoy [4], Bruno Albertini [1] and Antonio M. Saraiva [1,3]

Table S1. Dispersion of residual variance for each model. Significant dispersion models are highlighted in bold.

		Response	fixed-effects	dispersion	p-value
Replication	Perception	entrance	group + video quality	1.0036	0.872
			group	1.0028	0.868
			video quality	1.0018	0.86
			null	1.0009	0.86
		exit	group + video quality	0.86385	0.388
			group	0.86356	0.386
			video quality	0.86068	0.382
			null	0.86445	0.386
		pollen	group + video quality	1.0402	0.676
			group	1.0409	0.676
			video quality	1.0435	0.634
			null	1.0439	0.646
	Count	entrance	group + video quality	0.78575	0.896
			group	0.78227	0.916
			video quality	0.78299	0.906
			null	0.78214	0.93
		exit	group + video quality	0.48697	0.246
			group	0.48786	0.244
			video quality	0.48752	0.244
			null	0.48806	0.232
		pollen	group + video quality	1.4515	0.096
			group	1.4465	0.096
			video quality	1.4308	0.098
			null	1.426	0.124
Validation	Perception	entrance	group + video quality	1.05	0.838
			group	1.0487	0.838
			video quality	1.0497	0.846
			null	1.0481	0.858
		exit	group + video quality	0.94205	0.72
			group	0.94207	0.71
			video quality	0.94665	0.734
			null	0.94655	0.736
		pollen	group + video quality	1.0359	0.758
			group	1.0361	0.746
			video quality	1.0407	0.69
			null	1.0406	0.712
	Count	entrance	group + video quality	0.78728	0.934
			group	0.78883	0.912
			video quality	0.79104	0.924
			null	0.78844	0.932
		exit	group + video quality	0.47746	0.266
			group	0.47922	0.264
			video quality	0.47861	0.272
			null	0.47718	0.262
		pollen	**group + video quality**	**1.693**	**0.034**
			**group**	**1.6759**	**0.036**
			**video quality**	**1.7061**	**0.032**
			**null**	**1.6842**	**0.03**

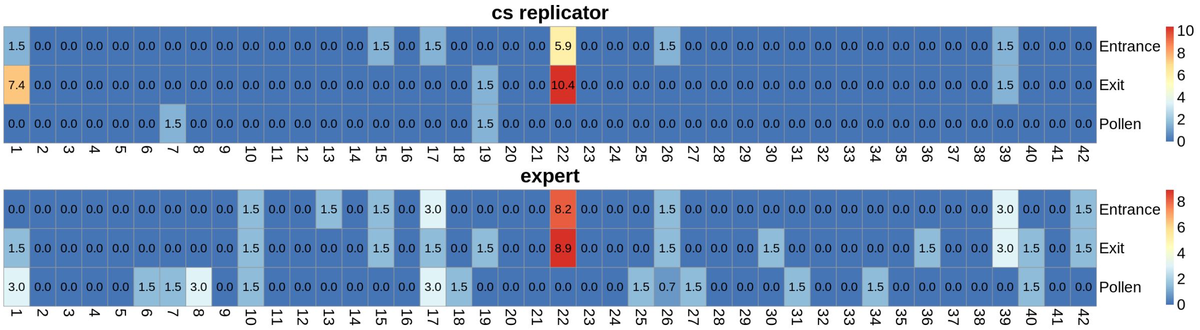

Figure S1. Median Absolute Deviation (MAD) value of each video for replicator citizen scientists and experts in different bee activities.
